# mPSQed: A Software for the Design of Multiplex Pyrosequencing Assays

**DOI:** 10.1371/journal.pone.0038140

**Published:** 2012-06-04

**Authors:** Piotr Wojtek Dabrowski, Andreas Nitsche

**Affiliations:** 1 Central Administration 4 (IT), Robert Koch Institute, Berlin, Germany; 2 Center for Biological Security 1, Robert Koch Institute, Berlin, Germany; J. Craig Venter Institute, United States of America

## Abstract

Molecular-based diagnostic assays are the gold standard for infectious diseases today, since they allow a rapid and sensitive identification and typing of various pathogens. While PCR can be designed to be specific for a certain pathogen, a subsequent sequence analysis is frequently required for confirmation or typing. The design of appropriate PCR-based assays is a complex task, especially when conserved discriminating polymorphisms are rare or if the number of types which need to be differentiated is high. One extremely useful but underused method for this purpose is the multiplex pyrosequencing technique. Unfortunately there is no software available to aid researchers in designing multiplex pyrosequencing assays. Here, we present mPSQed (Multiplex PyroSeQuencing EDitor), a program targeted at closing this gap. We also present the design of an exemplarily theoretical assay for the differentiation of human adenovirus types A–F using two pyrosequencing primers on two distinct PCR products, designed quickly and easily using our software.

## Introduction

Today the identification of infectious pathogens is usually based on the PCR-amplification and detection of stretches of the pathogen’s genome [1]. These stretches can be either selected to be highly pathogen-specific or to encompass a whole group, genus or family of pathogens. Particularly in this setting, a subsequent typing of the pathogen may be required for the completion of the diagnosis. With carefully designed probes, this can be achieved thanks to characteristic single nucleotide polymorphisms (SNPs) using methods such as fluorescence curve melting analysis [2]. Alternatively, the PCR product can be sequenced to obtain information on such polymorphisms. Since Sanger sequencing is well established but still comes along with some drawbacks, like the inability to sequence extremely short PCR amplicons often used in diagnostic PCR, and still some hours to obtain a result, pyrosequencing has evolved to be a promising alternative. In pyrosequencing, a linear amplification of the template is performed and the synthesis of the reverse complement strand can be monitored online. The reaction mix contains sulfurylase, luciferase, APS and luciferin in addition to classical PCR reagents. When a dNTP is incorporated into the strand, the resulting pyrophosphate is used by the sulfurylase to convert APS to ATP, which in turn provides the energy necessary for luciferase to generate light while converting luciferin to oxyluciferin. The resulting light signal is captured by a camera. In order to correlate the light signals created during the strand synthesis with specific bases, no dNTP mix is provided during the reaction. Instead, individual dNTPs are added to the reaction mix and then removed by addition of apyrase at pre-determined points of time. A recorded light signal can therefore be interpreted as proof for the incorporation of the currently present dNTP. The order in which light signals are detected in combination with the knowledge of the dNTP dispensation order can be used to reconstruct the exact sequence of the synthesized strand and thus also that of the template [3].

**Figure 1:**
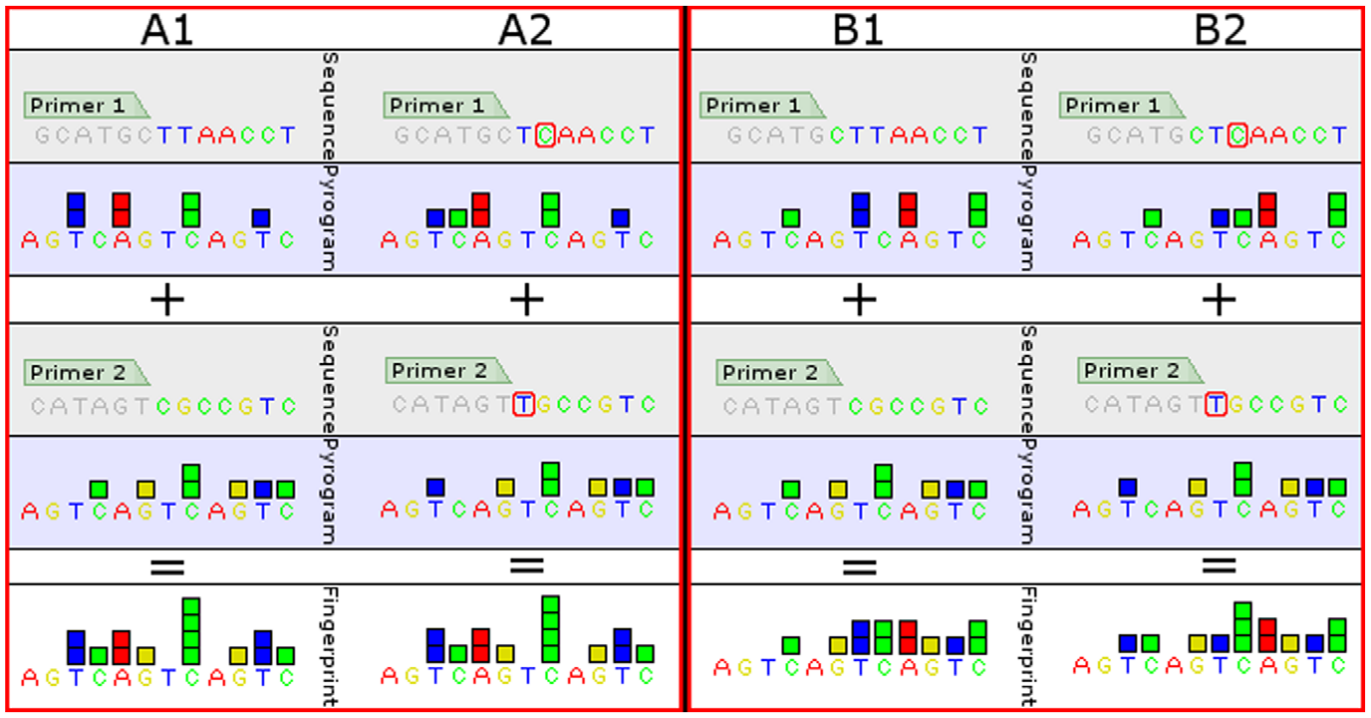
Principle of multiplex pyrosequencing. In multiplex pyrosequencing, several primers are used simultaneously in the sequencing reaction so that their signals overlap. A1: In this example, primer 1 (upper part) reads the sequence TTAACCT and primer 1 (middle part) reads the sequence CGCCGTC. Since the signals overlap, the fingerprint (lower part) represents the sequence TTCAAGCCCCGTTC. It is important to note that in this fingerprint, it is not possible to tell which base was read by which primer. A2: The T→C mutation after primer 1 and the C→T mutation after primer 2 are used as targets for differentiating between two species. However, they cancel each other out, causing the fingerprints for A1 and A2 to be identical. B: Moving primer 1 one base to the left alleviates this problem: the fingerprints for B1 and B2 are now different. This demonstrates the importance of correct pyrosequencing primer positioning relative to all utilized SNPs.

In contrast to Sanger sequencing, pyrosequencing only provides short sequence reads of up to 60 bases in regular runs, however, results can be obtained online subsequently to a completed PCR run in less than one hour. For any sequencing strategy, there are cases where no suitable stretches of sequence can be found that contain SNPs characteristic for all species of interest. To circumvent this problem, multiplex pyrosequencing can be used, wherein several PCR products with several pyrosequencing primers are sequenced simultaneously [4]. In this case, the signals created from each of the pyrosequencing primers overlap, creating a unique fingerprint pyrogram. While the fingerprint alone cannot be used to reconstruct the original sequences, it can be predicted given a priori knowledge of the expected sequences. Since each base in each of the sequences contributes to the final fingerprint, the presence of SNPs leads to different fingerprints for different species. Thus matching the pyrogram from a multiplex pyrosequencing experiment with a predicted fingerprint allows the identification of the corresponding species based on discriminating SNPs without explicit reconstruction of the underlying sequences (see Figure 1).

The design of primers for such multiplex pyrosequencing assays is a challenging task. Especially when reference genomes are large and numerous, the amount of data which needs to be considered can be overwhelming. Also, positioning pyrosequencing primers in a way which leads to unique fingerprints for each species of interest becomes harder with an increasing number of species. Since the fingerprint is a combination of signals from several sequences, incorrectly positioned pyrosequencing primers may lead to competing signals from SNPs in different sequences that cancel each other out (Figure 1). Manually calculating the fingerprints for many different primer positions is, while not infeasible, highly laborious and error-prone.

Currently, a wide range of nucleic acid sequence editors is available. One popular example is the free cross-platform sequence viewer SeaView that is geared towards multiple sequence alignment and phylogeny [5]. BioEdit, which is available for free for Windows, offers a wide range of tools for multiple sequence alignment, phylogeny, sequence analysis and interfaces to several online databases [6]. The cross-platform freeware MEGA offers an impressive array of tools for phylogenetic analysis [7]. In addition to free programs, many comparable commercial products such as Geneious [8], CLC Workbench [9] or Lasergene [10] exist. These programs focus on easy access to a large number of powerful analysis tools and visualisation of large datasets. Some of the mentioned programs integrate facilities for the design of primers and probes, be it through proprietary algorithms (as in the case of CLC Workbench) or through the integration of primer3 [11]. However, none of these programs offers support for either finding discriminating SNPs for diagnostic assays in general or for the design of multiplex pyrosequencing assays in particular.

Therefore, we have developed the mPSQed sequence editor which implements these aligned sequences can be grouped which allows the automatic identification of SNPs discriminating between groups. A tool for the design of multiplex pyrosequencing assays using the SNP information is further included. Thereby, multiplex pyrosequencing can be applied to a broader range of challenging diagnostic applications. In this publication we introduce the design ideas of mPSQed and give an introduction to its usage. Using the example of human adenovirus, we provide a step-by-step description of the analysis steps performed within mPSQed on the way from a sequence alignment to a complete discriminating multiplex pyrosequencing assay. Complete source code, binaries and all exemplary data are available from http://sourceforge.net/projects/mpsqed.

### Design and Implementation

In order to ensure cross-platform capability, the software was developed in Java. Thanks to Java’s extensive standard class libraries, this also allows online visualisation using only the language’s on-board capabilities. The online help system was designed using HelpSetMaker [12].

An open source license was chosen for mPSQed to allow all users to adapt the software to their specific wishes. As such, installation can take one of two routes: The source code can be downloaded from http://sourceforge.net/projects/mpsqed and compiled locally, or a runnable.jar archive can be obtained from the same address. The adenovirus alignment with annotated PCR and PSQ primers is also available on the sourceforge project page.

## Results and Discussion

**Figure 2:**
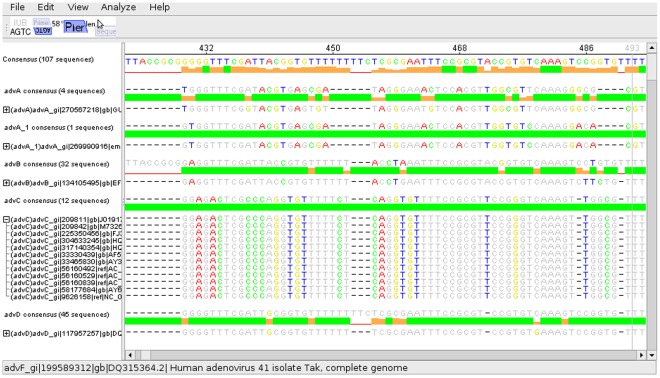
View of alignment with consensus sequence displayed for each group. Zoomed in view of an alignment where groups have been defined. The number of sequences which need to be displayed in order to capture the essential differences between the groups is significantly reduced (the five shown groups contain 94 sequences), but drilling down to the single sequence level is still easily possible, as visible in group “advC”. Bases which are identical to the consensus sequence (or reference sequence, which can be chosen manually) are gray, differing bases are colored based on the selected coloring scheme – “BioEdit” in this case.

**Figure 3:**
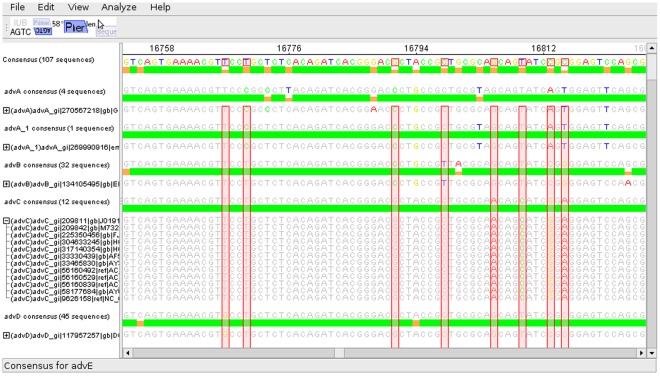
Display of SNPs which can be used to differentiate between groups. SNPs which can be used to differentiate between the defined groups must be perfectly conserved within each group (green column in the group’s consensus graph) and must differ between the groups (orange or red column in the global consensus graph at the top). These positions can be automatically identified and are marked by red columns in the alignment.

**Figure 4:**
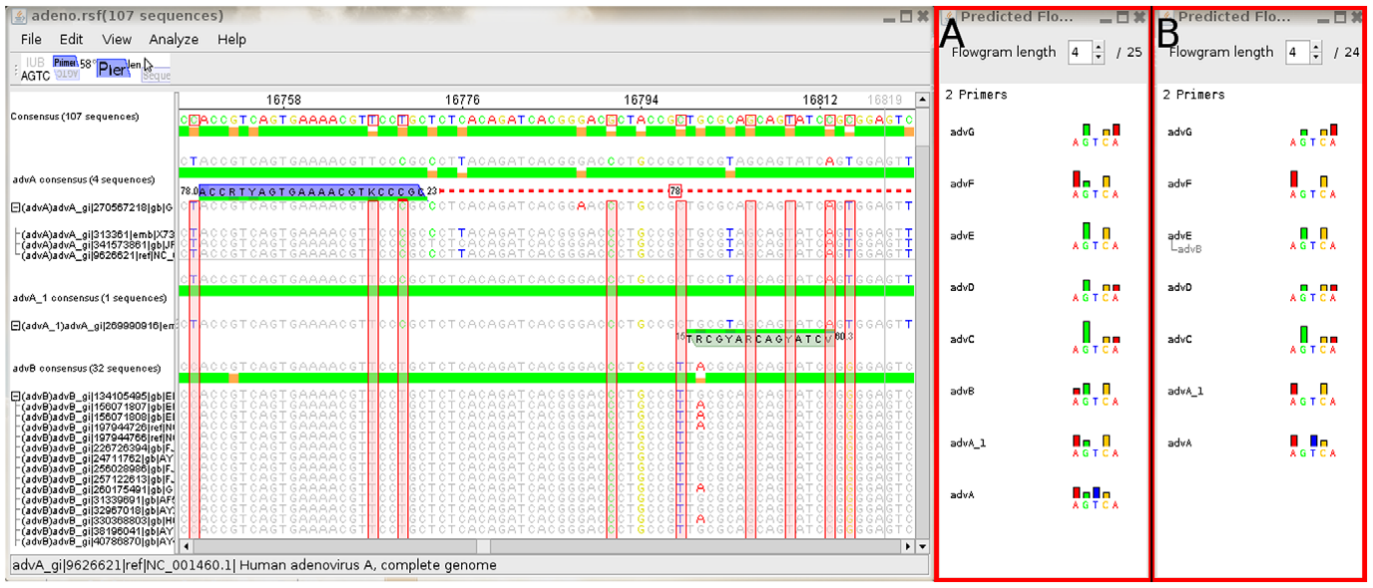
Design of multiplex pyrosequencing assay with display of predicted pyrograms. Display of an alignment with two pyrosequencing primers, one of which is visible on the screen (green annotation), and four PCR primers, one of which is also visible (blue annotation). For each primer, the melting temperature is displayed at the 5′ end and the length is displayed at the 3′ end. A line connects the forward PCR primer with its reverse counterpart (not visible, offscreen). The product size is shown in the middle of the connecting line, and the red color warns of a high difference in predicted melting temperature. In subfigure A, the predicted pyrograms from the two pyrosequencing primers are shown for each group – with just 5 cycles of the pyrosequencing machine, a unique pyrogram can be obtained for each of the groups. In subfigure B, the pyrosequencing primer has been moved one base to the left, thus preventing sequencing of one SNP. This leads to the predicted pyrograms for advE and advB being identical.

The identification of discriminating SNPs is an important step in the creation of diagnostic assays. To facilitate the selection of a set of discriminative SNPs several steps are required to be performed. After an alignment containing all relevant sequences has been loaded into the program the sequences can be grouped, and consensus sequences can be calculated both for the alignment globally and for each group individually. Groups can be collapsed, leaving only the conservation graph and the consensus sequence visible. This allows the user to work with a significantly reduced amount of visible data while still retaining easy access to all relevant information (see Figure 2). SNPs which are conserved within a single group and are thus candidates for use in a differentiation assay can be automatically detected and highlighted (see Figure 3). Some basic primer design functionality (such as Tm calculation, product size calculation, degeneration etc.) is supported. Novel functionality is provided for the design of multiplex pyrosequencing assays. Primers can be marked as pyrosequencing primers and the predicted pyrograms which would be generated using these primers in a multiplex pyrosequencing assay can be displayed. When pyrosequencing primers are moved, the predicted pyrograms are updated in realtime, allowing for a quick optimisation of primer positioning. If the expected pyrogram is not unique for each of the defined groups, a warning is displayed (see Figure 4).

To demonstrate the power of the described features, an exemplarily multiplex pyrosequencing assay has been designed which theoretically allows the identification of all human pathogenic adenoviruses (human adenovirus A to human adenovirus F). In this assay, two regions of the adenovirus are amplified using two sets of PCR primers, and a total of four SNPs across these amplicons is sequenced using two pyrosequencing primers. This allows the generation of a unique fingerprint for each of the mentioned adenovirus types within only five cycles of the pyrosequencing machine. To design this assay, all genomic sequences of these adenovirus types available from NCBI were aligned using mafft [13]. The alignment was loaded into the software, sequences were grouped according to type (Figure 2) and discriminating SNPs were automatically calculated (Figure 3). Then, regions with sufficiently high conservation were determined and pyrosequencing primers were positioned so as to allow the sequencing of several SNPs. Finally, the position of the pyrosequencing primers was manually optimized using the realtime prediction of pyrograms and PCR primers were added. These steps are illustrated in (Figure 4), with the predicted pyrograms for all adenovirus types shown in Figure 4A. Once the alignment was calculated, the whole design process took approximately 30 minutes on a regular desktop computer. This demonstrates the usefulness of this software in the otherwise complex task of designing multiplex pyrosequencing assays. To allow novice users easy access to this functionality, an extensive online help describing all features is available from within the program.

The alignment which was used in the creation of the assay, including annotations for both PCR and pyrosequencing primers, is available for download as supporting information.

### Availability and Future Directions

Precompiled binaries for Windows, Linux and Mac computers and source code for the software can be obtained from http://sourceforge.net/projects/mpsqed without any restrictions. The binaries are provided as a zip file containing a runnable jar file and all necessary libraries. We also provide the data used in this contribution to allow users to follow through this example analysis. Further development will now focus on extending the tools for primer design, such as providing Tm graphs for the sequences, secondary structure prediction and the prediction of primer-primer interactions. Furthermore, functionality for automatic assay design is planned: Since discriminating SNPs are already automatically identified, sets of SNPs which could be used together in an assay and are located close to conserved regions suited for primers should also be automatically determined and presented to the user as suggestions.

Since the software is open-source, modifications can be made by anyone. The easiest and most practical place is likely the extension of supported file formats. By implementing the interfaces LoadFilter and SaveFilter in the package org.rki.sequenceeditor.model.filters, capability to read and/or write arbitrary file formats can easily be added to the program.
